# Description, molecular characteristics and *Wolbachia* endosymbionts of *Onchocerca borneensis* Uni, Mat Udin & Takaoka n. sp. (Nematoda: Filarioidea) from the Bornean bearded pig *Sus barbatus* Müller (Cetartiodactyla: Suidae) of Sarawak, Malaysia

**DOI:** 10.1186/s13071-020-3907-8

**Published:** 2020-02-06

**Authors:** Shigehiko Uni, Ahmad Syihan Mat Udin, Takeshi Agatsuma, Kerstin Junker, Weerachai Saijuntha, Naruemon Bunchom, Masako Fukuda, Coralie Martin, Emilie Lefoulon, Amandine Labat, Faisal Ali Anwarali Khan, Van Lun Low, Phaik Leng Cheah, Yvonne Ai-Lian Lim, Rosli Ramli, Daicus Martin Belabut, Nur Afiqah Zainuri, Makoto Matsubayashi, Hasmahzaiti Omar, Subha Bhassu, Shoji Uga, Rosli Hashim, Hiroyuki Takaoka, Mohd Sofian Azirun

**Affiliations:** 1 Institute of Biological Sciences, Faculty of Science, University of Malaya, 50603 Kuala Lumpur, Malaysia; 2 Department of Public Health, Faculty of Nursing, Kobe Women’s University, Kobe, 650-0046 Japan; 3 Department of Environmental Medicine, Kochi Medical School, Kochi University, Nankoku, 783-8505 Japan; 4 ARC-Onderstepoort Veterinary Institute, Private Bag X05, Onderstepoort, 0110 South Africa; 5 Walai Rukhavej Botanical Research Institute, Mahasarakham University, Maha Sarakham, 44150 Thailand; 6 Institute for Research Promotion, Oita University, Oita, 879-5593 Japan; 7 UMR7245, MCAM, Muséum National d’Histoire Naturelle, 75005 Paris, France; 8 Molecular Parasitology Group, New England Biolabs, Inc, Ipswich, MA 01938 USA; 9 Department of Zoology, Faculty of Resource Sciences and Technology, Universiti Malaysia Sarawak, 943800 Kota Samarahan, Sarawak Malaysia; 10 Tropical Infectious Diseases Research & Education Centre, University of Malaya, 50603 Kuala Lumpur, Malaysia; 11 Department of Pathology, Faculty of Medicine, University of Malaya, 50603 Kuala Lumpur, Malaysia; 12 Department of Parasitology, Faculty of Medicine, University of Malaya, 50603 Kuala Lumpur, Malaysia; 13 Department of International Prevention of Epidemics, Division of Veterinary Science, Graduate School of Life and Environmental Sciences, Osaka Prefecture University, Osaka, 598-8531 Japan; 14 Centre for Biotechnology in Agriculture, CEBAR, University of Malaya, 50300 Kuala Lumpur, Malaysia

**Keywords:** Coevolution, Indomalayan realm, *Malayfilaria sofiani*, *Onchocerca dewittei*, *Onchocerca japonica*, Suidae

## Abstract

**Background:**

The genus *Onchocerca* Diesing, 1841 includes species of medical importance, such as *O. volvulus* (Leuckart, 1893), which causes river blindness in the tropics. Recently, zoonotic onchocercosis has been reported in humans worldwide. In Japan, *O. dewittei japonica* Uni, Bain & Takaoka, 2001 from wild boars is a causative agent for this zoonosis. Many filarioid nematodes are infected with *Wolbachia* endosymbionts which exhibit various evolutionary relationships with their hosts. While investigating the filarial fauna of Borneo, we discovered an undescribed *Onchocerca* species in the bearded pig *Sus barbatus* Müller (Cetartiodactyla: Suidae).

**Methods:**

We isolated *Onchocerca* specimens from bearded pigs and examined their morphology. For comparative material, we collected fresh specimens of *O. d. dewittei* Bain, Ramachandran, Petter & Mak, 1977 from banded pigs (*S. scrofa vittatus* Boie) in Peninsular Malaysia. Partial sequences of three different genes (two mitochondrial genes, *cox*1 and *12S* rRNA, and one nuclear ITS region) of these filarioids were analysed. By multi-locus sequence analyses based on six genes (*16S* rDNA, *ftsZ*, *dnaA*, *coxA*, *fbpA* and *gatB*) of *Wolbachia*, we determined the supergroups in the specimens from bearded pigs and those of *O. d. dewittei*.

**Results:**

*Onchocerca borneensis* Uni, Mat Udin & Takaoka n. sp. is described on the basis of morphological characteristics and its genetic divergence from congeners. Molecular characteristics of the new species revealed its close evolutionary relationship with *O. d. dewittei*. Calculated p-distance for the *cox*1 gene sequences between *O. borneensis* n. sp. and *O. d. dewittei* was 5.9%, while that between *O. d. dewittei* and *O. d. japonica* was 7.6%. No intraspecific genetic variation was found for the new species. *Wolbachia* strains identified in the new species and *O. d. dewittei* belonged to supergroup C and are closely related.

**Conclusions:**

Our molecular analyses of filarioids from Asian suids indicate that the new species is sister to *O. d. dewittei*. On the basis of its morphological and molecular characteristics, we propose to elevate *O. d. japonica* to species level as *O. japonica* Uni, Bain & Takaoka, 2001. Coevolutionary relationships exist between the *Wolbachia* strains and their filarial hosts in Borneo and Peninsular Malaysia.
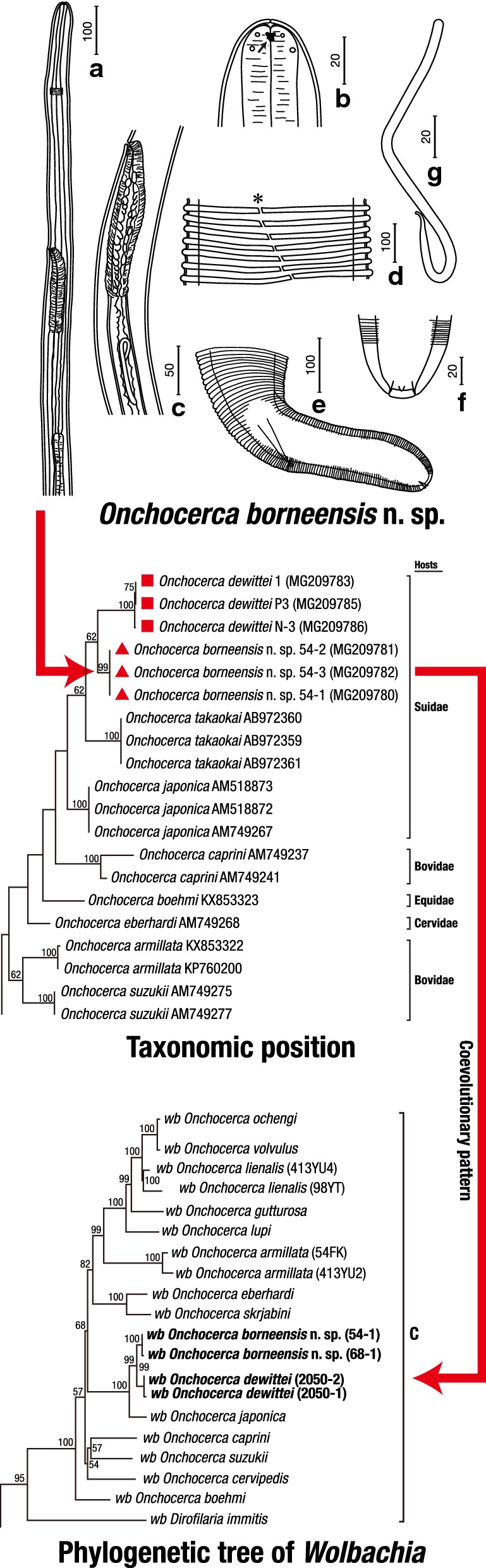

## Background

The genus *Onchocerca* Diesing, 1841 (Onchocercidae) is well known for its medical importance, with *O. volvulus* (Leuckart, 1893) causing river blindness or onchocercosis in humans. In 2018, the World Health Organization listed onchocercosis as a major cause of ocular and skin diseases in sub-Saharan Africa and parts of Latin America [1]. Recently, zoonotic infections with *Onchocerca* spp. have been reported in humans worldwide. *Onchocerca lupi* Rodonaja, 1967 from wolves, dogs and cats has been reported to cause lesions in the subconjunctival region, skin or spinal cord in humans in the USA, Turkey, Tunisia, Iran and Germany [2–4]. In Japan, zoonotic cases owing to *O. dewittei japonica* Uni, Bain & Takaoka, 2001 from the Japanese wild boar *Sus scrofa leucomystax* Temminck (Suidae) have been documented [5–8]. In other zoonotic cases, the causative agents have been identified as *O. gutturosa* Neumann, 1910 from cattle, *O. cervicalis* Railliet & Henry, 1910 from horses and *O. jakutensis* (Gubanov, 1964) from European roe deer [9–11]. In order to assess the zoonotic potential of *Onchocerca* spp., it is necessary to better define the biodiversity within the genus, and their epidemiology in their domestic or wild hosts throughout their geographic range [12, 13].

To date, 33 species of *Onchocerca* and one subspecies (*O. d. japonica*) have been recorded worldwide [6, 14–18]. According to Lefoulon et al. [19], multi-locus sequence analyses suggest a division of members of the Onchocercidae into five clades (ONC1–ONC5), with ONC3 containing three genera, namely *Onchocerca*, *Loxodontofilaria* Berghe & Gillain, 1939 and *Dirofilaria* Railliet & Henry, 1910. Moreover, members of the genus *Onchocerca* are divided into three clades: *O*. *d. japonica* from the Suidae is placed in the first clade, which is the most diverse with regard to the host range [20, 21]. Although 35 species of filarial parasites from 22 genera have previously been recorded in vertebrates in Malaysia [15, 22–24], *O. dewittei* Bain, Ramachandran, Petter & Mak, 1977 is the only species of *Onchocerca* reported there. However, the origin, molecular characteristics and evolutionary relationships of *O. dewittei* remain to be clarified [25–27].

While participating in the Heart of Borneo Scientific Expedition organized by the Department of Forestry, Sarawak in 2016, we found an undescribed *Onchocerca* species in the bearded pig *S. barbatus* Müller in Long Banga, Sarawak. To elucidate the diversity and molecular relationships of *Onchocerca* spp. from suid hosts in Asia, we compared the specimens isolated from Bornean bearded pigs with specimens of *O. d. dewittei* recently collected from the banded pig *S. s. vittatus* Boie in Peninsular Malaysia and with those of *O. d. japonica* previously obtained from wild boars in Japan. Our results indicate that the specimens from Borneo differ from their congeners at the species level but are closely related to *O. d. dewittei*. In addition, our molecular analyses proved that *O. d. dewittei* and *O. d. japonica* are distinct species.

*Wolbachia* endosymbionts are alpha-proteobacteria widely distributed in arthropods and filarioid nematodes [28–33]. *Wolbachia* strains infect the female germline, lateral hypodermal chords and intestinal wall cells in many species in the family Onchocercidae [34, 35]. The endosymbionts are essential for the fertility and growth of their filarial hosts and exhibit various evolutionary relationships with them [28, 30, 31, 34, 35]. We examined *Wolbachia* endosymbionts in the specimens from Bornean bearded pigs and those of *O. d. dewittei* from banded pigs in Peninsular Malaysia. The *Wolbachia* supergroups were identified by multi-locus sequence analyses [19, 28]. On the basis of our findings, we discuss the molecular relationships between *Wolbachia* strains and their *Onchocerca* hosts in Asia. Furthermore, to compare *Wolbachia* strains of other filarial nematodes obtained in Malaysia with *Wolbachia* strains of the present *Onchocerca* spp., we detected a *Wolbachia* strain in *Malayfilaria sofiani* Uni, Mat Udin & Takaoka, 2017 (Onchocercidae), previously isolated from the common treeshrew *Tupaia glis* Diard & Duvaucel (Mammalia: Scandentia) in Peninsular Malaysia [24]. We discuss the molecular relationships between *Wolbachia* strains and their onchocercid hosts *M. sofiani*, *Wuchereria bancrofti* (Cobbold, 1877) and species of *Brugia* Buckley, 1958.

## Methods

### Collection of hosts and parasites

The Heart of Borneo Scientific Expedition organized by the Department of Forestry in Long Banga (3°12′0″N, 115°22′59.99″E), Sarawak, Malaysia, was undertaken between 20 August 2016 and 2 September 2016. During the expedition, the feet (*n* = 12) of three Bornean bearded pigs (ID nos. WB30, WB54 and WB68), legally captured by local inhabitants, were examined. Filarial parasites obtained from the footpads of the bearded pigs (ID nos. WB54 and WB68) were subjected to subsequent morphological and molecular studies, and pieces of muscle obtained from these two bearded pigs were used for molecular identification of the host species.

### Morphological methods

To search for parasites, the footpads were dissected under a stereomicroscope. Isolated adult worms were fixed in 70% ethanol for morphological examination. Worms were cleared in lactophenol (R & M Chemicals, Essex, UK) and drawn under a compound microscope equipped with a camera lucida (Olympus U-DA, Olympus, Tokyo, Japan). The mid-region of a fixed female was embedded in paraffin, and sections were stained with haematoxylin and eosin (HE). Skin snips were taken from the limbs of the infected bearded pigs to examine microfilariae [36]. Thick blood smears were made and stained with 3% Giemsa solution (pH 7.4). Skin snips and blood smears were examined for microfilariae under a compound microscope.

### Comparative material examined

#### *Onchocerca dewittei dewittei* collected from banded pigs in Peninsular Malaysia

Five banded pigs (ID nos. WP1-WP5) from Peninsular Malaysia were examined for filarial parasites. Because of the absence of a beard, these animals were identified as banded pigs. Three of the five animals (ID nos. WP1, WP4 and WP5) were legally captured by local inhabitants at the Field Studies Center (3°19′29.0″N, 101°45′09.7″E) of the University of Malaya, situated in the primary forest of Ulu Gombak, Selangor, on 27 February 2012, 14 December 2012 and 20 May 2016, respectively. Two of the banded pigs (ID nos. WP2 and WP3) were captured on an oil palm plantation at Sungai Besar (3°42′11.8″N, 101°05′53.2″E), Sabak Bernam District, Selangor, on 7 April 2012 and 14 October 2012, respectively. The two animals were provided by local residents with the permission of the oil palm plantation owner. A piece of muscle of one banded pig (ID no. WP5), captured at Ulu Gombak, was used for molecular identification of the host species. Skin snips were taken from the limbs of the banded pigs to examine microfilariae. Adult worms were obtained from the footpads of the banded pigs and examined as described above. Skin snips and blood smears were examined for microfilariae.

#### *Onchocerca dewittei japonica* obtained from the wild boar in Japan

Seven adults (four fragments of females and three fragments of males) of *O. d. japonica* had been collected from the footpads of two Japanese wild boars (ID nos. OB4 and OB5) in Bungoono (32°58′41.1″N, 131°35′06.2″E), Oita, Kyushu, Japan, on 7 November 2011. Six specimens were fixed in 70% ethanol and examined as described above. One female fragment, fixed in 80% ethanol, was used for the present molecular analysis.

#### *Malayfilaria sofiani* isolated from the common treeshrew in Peninsular Malaysia

One fragment of a female of *M. sofiani* (ID no. KE-2), fixed in 80% ethanol, was used to determine *Wolbachia* and its supergroup affiliation [24].

### Molecular analysis of filarioid nematodes and host animals

The following materials were transferred directly into 80% ethanol and used for molecular analyses: Four *Onchocerca* females (ID nos. 54-1, 54-2, 54-3 and 68-1) collected from bearded pigs (ID nos. WB54 and WB68) in Borneo; seven fragments of females (ID nos. 1, N2-1, N-3, No3, No4, P-1 and P3) of *O. d. dewittei* obtained from banded pigs (ID nos. WP1, WP4 and WP5) in Peninsular Malaysia; one fragment of a female of *O. d. japonica* (ID no. 1; see above) in Japan; microfilariae obtained from the skin snips from a banded pig (ID no. WP5); pieces of muscle from limbs of the bearded pigs (ID nos. WB54, WB68) and a banded pig (WP5).

In order to determine the partial sequences of the mitochondrial *cox*1 and *12S* rRNA genes of *Onchocerca* specimens, DNA extraction, polymerase chain reaction (PCR) amplification, and sequencing were performed as described previously [7, 24, 37, 38]. *Filaria martis* Gmelin, 1790 (Filariidae) was selected as the outgroup for the phylogenetic analyses based on the *cox*1 and *12S* rRNA gene sequences. We also cloned the PCR products of the nuclear ITS region into pGEM-T vectors and determined the sequences of the recombinant plasmid [39]. *Wuchereria bancrofti* and *B. malayi* (Brug, 1927) were selected as outgroups for the ITS region. In addition, we determined the sequences of the cytochrome *b* gene (*cyt*b) of bearded pigs in Borneo and a banded pig in Ulu Gombak, Peninsular Malaysia, following the protocol of Watanobe et al. [40].

The newly generated sequences were deposited in the GenBank database. GenBank accession numbers of the new sequences and those of other filarioid nematodes and Suidae used to compare the present specimens to are provided in the figures. We calculated uncorrected p-distances between species of filarial parasites in MEGA7 as an estimate of the accumulated number of nucleotide substitutions per site [41]. Phylogenetic trees of the nucleotide sequences of the *cox*1 and *12S* rRNA genes and the ITS region of *Onchocerca* spp. and the *cyt*b gene of the host animals were constructed using the maximum-likelihood (ML) method in MEGA7 [41], with 500 bootstrap replicates. The lengths of the sequence datasets used for the analyses were as follows: *cox*1, 393 bp; *12S* rRNA, 304 bp; ITS, 866 bp; and *cyt*b, 741 bp.

### *Wolbachia* detection methods

#### Immunohistochemical staining

Sections of a female worm of *O. d. dewittei* were stained with a rabbit polyclonal antiserum raised against the surface protein of *Wolbachia* from *B*. *pahangi* (Buckley & Edeson, 1956) (1:2000 dilution), as described by Kramer et al. [42] and Ferri et al. [34].

#### Molecular screening

DNA was extracted from two female fragments (ID nos. 54-1 and 68-1) of *Onchocerca* specimens obtained from Bornean bearded pigs (ID nos. WB54 and WB68, respectively), two fragments of females (ID nos. 2050-1 and 2050-2) of *O. d. dewittei* obtained from a banded pig (ID no. WP5), and one fragment of a female (ID no. KE-2) of *M. sofiani* from a common treeshrew in Peninsular Malaysia, using the QIAamp^®^ DNA Mini kit, following the protocol “DNA purification from tissues” recommended by the manufacturer (Qiagen, Courtaboef, France). *Wolbachia* symbionts were determined by nested PCR screening of the six genes (*16S* rDNA, *ftsZ*, *dnaA*, *coxA*, *fbpA* and *gatB*), as described by Lefoulon et al. [19, 28, 35]. PCR products were purified and sequenced by Eurofins Genomics. Supergroups of *Wolbachia* were identified as described by Lo et al. [43] and Lefoulon et al. [28].

#### Phylogenetic analysis

The sequences of the six genes of *Wolbachia* were aligned with sequences available in GenBank (Additional file 3: Table S2) using MAFFT [44]. For the nucleotide supermatrix, the phylogeny of *Wolbachia* strains was performed by ML inference using TIM + F + I + G4 with IQ-TREE version 1.5 [45, 46]. The robustness of nodes was assessed with 1000 bootstrap replicates. The length of the supermatrix dataset was 3086 bp.

## Results

**Family Onchocercidae Leiper, 1911**

**Subfamily Onchocercinae Leiper, 1911**

***Onchocerca borneensis*** **Uni, Mat Udin & Takaoka n. sp.**

***Type-host***: *Sus barbatus* Müller (Cetartiodactyla: Suidae), Bornean bearded pig.

***Type-locality***: Long Banga (3°12′0″N, 115°22′59.99″E), Ulu Baram, Sarawak, Malaysia.

***Type-material***: Holotype female (MNHN 103YT) and allotype male (MNHN 104YT) were deposited in the Muséum National d’Histoire Naturelle, Paris, France. Paratypes (7 females: Ob-B54-1–2, Ob-B54-4, Ob-B54-6–7, Ob-54-9, Ob-B68-2; 10 males: Ob-B54-2M1–2, Ob-B54-3M1, and Ob-B68-2-M3–9) were deposited in the Institute of Biological Sciences, University of Malaya, Malaysia. Collection dates: 31.viii.2016 and 1.ix.2016.

***Site in host***: Adult worms were found in nodular fibrous structures in the adipose tissue of footpads of fore- and hindlimbs.

***Prevalence and intensity of infection***: Two of three bearded pigs were infected with adult worms: seven females and four males in the bearded pig WB54, and one female and seven males in the bearded pig WB68.

***Representative DNA sequences***: Sequence data were deposited in the GenBank database as follows: *cox*1 (MG209780-MG209782), *12S* rRNA gene (MG209790-MG209792) and ITS (MG192125-MG192127) for *O. borneensis* n. sp.; *cyt*b (MG657264-MG657265) for *S. barbatus*. Accession numbers of *Wolbachia* sequences are provided in Additional file 3: Table S2.

***ZooBank registration***: To comply with the regulations set out in article 8.5 of the amended 2012 version of the *International Code of Zoological Nomenclature* (ICZN) [47], details of the new species have been submitted to ZooBank. The Life Science Identifier (LSID) of the article is urn:lsid:zoobank.org:pub: F33A99AB-EDB5-40BF-BFBB-34033F4FF1CF. The LSID for the new name *Onchocerca borneensis* Uni, Mat Udin & Takaoka n. sp. is urn:lsid:zoobank.org:act: 018F7D26-7650-46AF-9330-4826C46CD53B.

***Etymology***: The specific epithet is derived from Borneo Island, the location where the type-host was collected.

**Description**

**Fig. 1:**
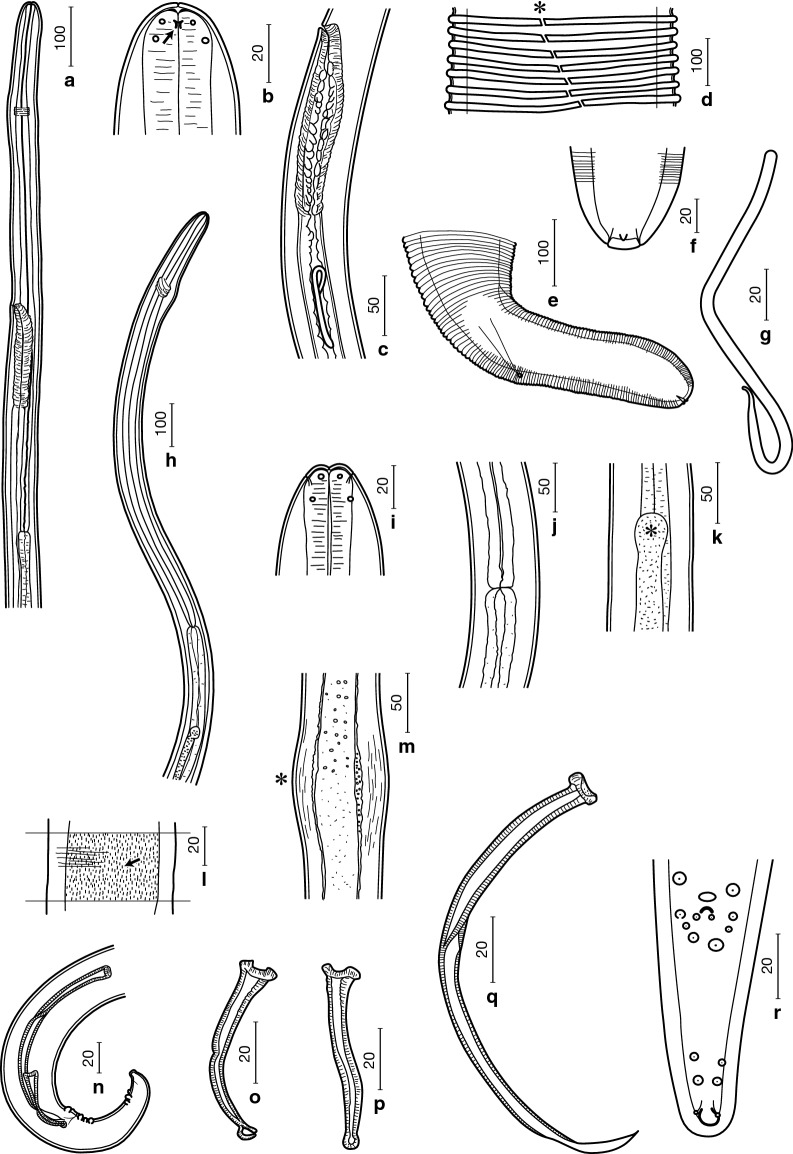
Line drawings of *Onchocerca borneensis* n. sp. Females (**a**–**f**), microfilaria (**g**) and males (**h**–**r**). **a** Anterior end, left lateral view. **b** Anterior extremity, lateral view, showing amphid (arrow). **c** Vagina, left lateral view. **d** Transverse cuticular ridges at midbody region, showing lateral field (*). **e** Posterior end, left lateral view. **f** Posterior extremity, ventral view, showing internal terminal point and two subterminal phasmids. **g** Microfilaria without sheath. **h** Anterior end, lateral view. **i** Anterior extremity, dorsoventral view. **j** Oesophago-intestinal junction. **k** Apex of testis (*). **l** Short longitudinal cuticular crests (arrow) at midbody region. **m** Body swelling (*). **n** Posterior end, right lateral view. **o** Right spicule, lateral view. **p** Right spicule, dorsoventral view. **q** Left spicule, lateral view. **r** Posterior end, ventral view. *Scale-bars* are in micrometres

**Fig. 2:**
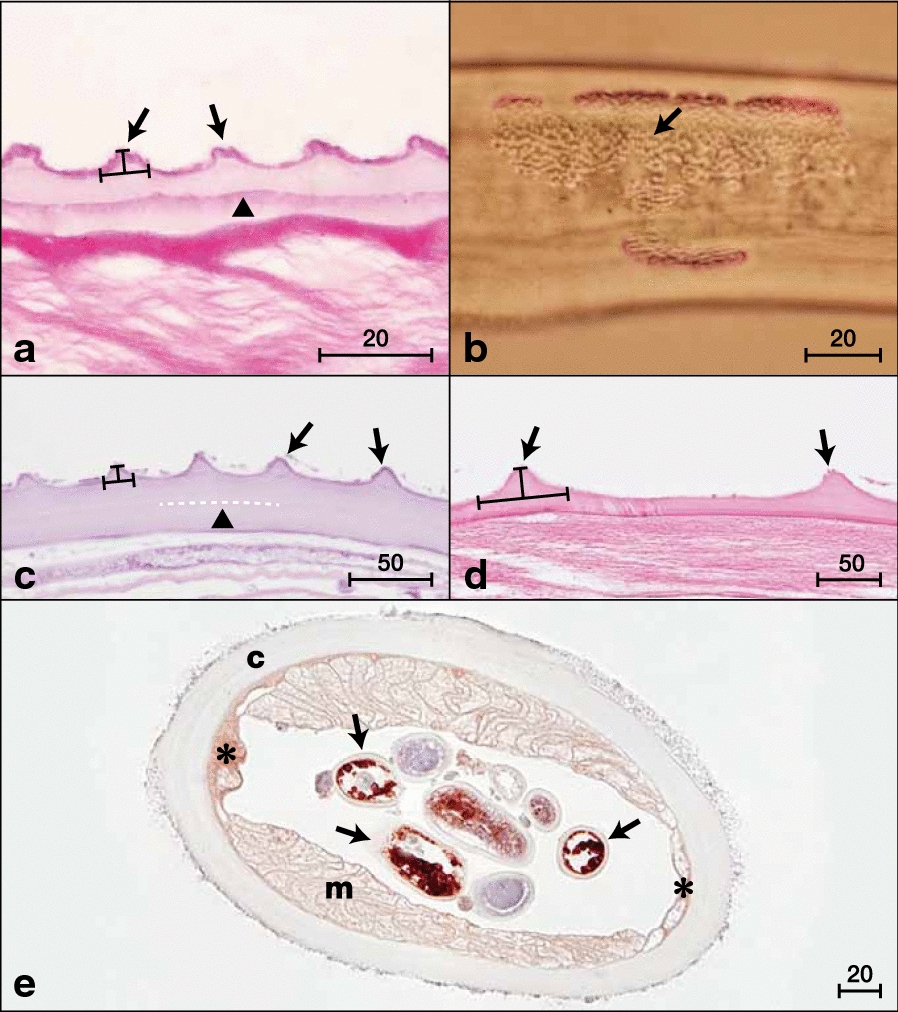
Light micrographs of *Onchocerca borneensis* n. sp. (**a** and **b**), *O. dewittei dewittei* (**c** and **e**) and *O. d. japonica* (**d**). **a** Longitudinal section of female at midbody: transverse cuticular ridges, triangular or trapezoid in shape (arrows; height and width, dark lines) and absence of internal striae at mid-line (arrowhead) (HE staining). **b** Short longitudinal cuticular crests (arrow) of male at midbody. **c** Longitudinal section of female at midbody: triangular transverse cuticular ridges (arrows; height and width, dark lines) and absence of internal striae (arrowhead) at mid-line (dotted line) (HE staining). **d** Longitudinal section of female at midbody: large triangular transverse cuticular ridges (arrows; height and width, dark lines), showing long distance between adjacent ridges (HE staining). **e** Transverse section of female with *Wolbachia* immunostaining. Dark red dots in ovaries and uterus (arrows) are *Wolbachia*. Lateral chords (*), muscles (m) and cuticle (c). *Scale-bars* are in micrometres

***General.*** [Table 1; Figs. 1, 2]. Body slender, tapering towards both extremities. Anterior extremity very thin, straight, with rounded apex (Fig. 1a). Labial and cephalic papillae arranged in a circle of 4 each (Fig. 1b). Amphids lateral, on level of labial papillae. Mouth opening small; thin sclerotized lamella present between head cuticle and oesophageal apex (Fig. 1b). Oesophagus not divided, anterior portion thin, gradually widening posteriorly. Nerve-ring surrounding oesophagus on level of anterior portion. Slight cervical swelling present at nerve-ring. Deirids and excretory pore not observed. Body cuticle of females with external transverse ridges (Fig. 1d). Caudal papillae in males prominent, grouped near cloaca (Fig. 1r). Microfilaria unsheathed (Fig. 1g).

**Table 1:** Comparative morphometric data for *Onchocerca borneensis* n. sp. and congeners recorded from wild suids

Species	*Onchocerca borneensis* n. sp.^a^	*Onchocerca dewittei dewittei* Bain, Ramachandran, Petter & Mak, 1977	*Onchocerca dewittei japonica* Uni, Bain & Takaoka, 2001	*Onchocerca takaokai* Uni, Fukuda & Bain, 2015^d^	*Onchocerca ramachandrini* Bain, Wahl & Renz, 1993
Reference	Present study	Present study	Present study	[18]	[16]
Host	*Sus barbatus*	*Sus scrofa vittatus*	*Sus scrofa leucomystax*	*Sus scrofa leucomystax*	*Phacochoerus africanus*
Locality	Long Banga, Sarawak, Malaysia	Ulu Gombak, Selangor, Malaysia	Bungoono, Oita, Japan	Oita, Japan	Cameroon
Female	(*n* = 8)	(*n* = 14)	(*n* = 3)	Holotype	Holotype
Body length (cm)	21.5 (17.5–21.5)	30.7^b^	16.7–27.3^c^	–	12.8
Body width at midbody	490 (410–590)	220–320	260–430	110	210
Nerve-ring from anterior end	193 (170–225)	195–200	207–233	190	250
Oesophagus length	975 (900–1288)	988–1438	1020–1120	1270	1250
Vulva from anterior end	513 (475–680)	343–497	520–620	475	650
Distance of ridges at midbody	17 (10–25)	40–93	235–340	Ridges absent	Longitudinal crests
Ridges (height/width) at midbody	5 (4–5)/10 (9–10)	10–11/15–20	23–35/75–100	–	–
Cuticle thickness at midbody	17 (10–36)	20–40	13–33	8	15–20
Tail length	258 (183–258)	143–188	100–218	130	170
Microfilaria	(*n* = 10)	(*n* = 10)	(*n* = 10)	(*n* = 4)	(*n* = 11)
Body length	160–188	198–245	158–203	295–329	290–325
Body width	5–6	5–8	5–6	6–9	7.0–7.5
Male	(*n* = 11)	(*n* = 2)	(*n* = 3)	–	(*n* = 2)
Body length (mm)	18 (16–24)	42–45	51–54^c^	–	32.3–34
Body width at midbody	65 (50–110)	88–93	100–113	–	65–72
Cuticular crests at midbody	Present	Present	Present	–	None
Nerve-ring from anterior end	225 (163–225)	213	167–247	–	175–190
Oesophagus length	1040 (800–1070)	905	888–1007	–	1000–1050
Right spicule length (RS)	63 (58–85)	75–88	73–77	–	78
Left spicule length (LS)	163 (125–198)	223–245	223–250	–	220–240
Spicule length ratio (LS/RS)	2.6 (2.1–2.8)	2.8–3.0	3.1–3.3	–	2.9
Tail length	70 (63–80)	53–63	62–80	–	105–125
Parasitic location of adults	Footpads	Footpads	Footpads	Skin of head, neck and back	Subcutaneous connective tissues of feet

^a^Measurements of the holotype female and allotype male of *Onchocerca borneensis* n. sp. are presented first, followed by the range, including the type-specimen, in parentheses

^b^[15]

^c^[6]

^d^Males not yet found

*Note*: Measurements are in micrometres unless otherwise stated

***Female.*** [Based on the holotype, 3 complete worms and 4 fragmented specimens; Table 1, Figs. 1a–f, 2a.] Vulva at mid-level of oesophagus (Fig. 1a). Vagina straight, simple (Fig. 1c). Ovejector straight, parallel to oesophagus, posteriorly directed. Uterus didelphic and opisthodelphic. Body distinctly widened over much of its length (Table 1). Cuticle with fine external transverse ridges at 1.6 mm from anterior extremity, more pronounced posteriorly. Transverse ridges straight, interrupted in lateral fields, without bifurcations (Fig. 1d). In longitudinal sections at midbody, ridges triangular or trapezoid in shape, with rounded or flattened tips. Median layer of cuticle without internal striae (Fig. 2a). Tail bent dorsally (Fig. 1e), tail extremity with internal terminal point and 2 subterminal phasmids (Fig. 1f).

***Male.*** [Based on the allotype and 10 complete specimens; Table 1, Figs. 1h–r, 2b.] Anterior extremity attenuated, rounded (Fig. 1h). Apex of testis 1.6–2.7 mm from anterior extremity, posterior to oesophago-intestinal junction (Fig. 1k). Main part of body with short longitudinal cuticular crests (Figs. 1l, 2b). In anterior half of body, 2 to 4 body swellings with pseudocoelomocytes (Fig. 1m). In one male (16 mm long), three body swellings at 2.5 mm, 4.8 mm and 7.4 mm from anterior extremity. *Area rugosa* absent. Right spicule with dent in mid-section and knobbed distal end in lateral view (Fig. 1o). Left spicule divided into handle and lamina; lamina with attenuated long membranous extremity (Fig. 1q). Gubernaculum absent. Narrow caudal alae present. Caudal papillae 7 pairs, arranged in 2 groups: 4 paracloacal sublateral pairs and 1 small subventral postcloacal pair; and 2 sublateral pairs in posterior third of tail. One unpaired precloacal ventral papilla present anterior to cloaca. Tail bent ventrally. Posterior extremity with large subcuticular knob and phasmids at its base (Fig. 1r).

***Microfilaria.*** [Based on 10 specimens from uterus of a fixed female; Table 1, Fig. 1g.] Body 160–188 long, 5–6 wide, unsheathed. Anterior extremity rounded, tail extremity attenuated to curled blunt point.

**Remarks**

The present specimens were assigned to the genus *Onchocerca* as defined by Anderson & Bain [48]: preoesophageal ring absent; anterior extremity rounded; body not markedly tapering anteriorly and posteriorly in female; body cuticle of females with transverse ridges; and caudal papillae in males prominent, grouped near cloaca.

We thus compared the morphological characteristics of the present specimens with those of the 33 species and a single subspecies (*O. d. japonica*) currently included in the genus [6, 14, 17, 18, 20, 49]. Females of many *Onchocerca* spp. possess transverse cuticular ridges and internal cuticular striae [14, 17]. However, *O. borneensis* n. sp. closely resembles its congeners described from suid hosts, *O. d. dewittei* in Peninsular Malaysia and *O. d. japonica* in Japan, in having transverse cuticular ridges but lacking internal striae in females (Figs. 1d, 2a, c, d). The new species, *O. d. dewittei* and *O. d. japonica* possess short longitudinal crests in males (Figs. 1l, 2b) [6]. The new species is distinct from *O. d. dewittei* and *O. d. japonica* in that females are wider at midbody and have a longer tail, as well as that males are shorter, only reaching half the length of males of the latter two species, and have a shorter left spicule (Table 1). The distance between two adjacent cuticular ridges at midbody in females of *O. borneensis* n. sp. is one-third that of *O. d. dewittei* and less than one-tenth that of *O. d. japonica*. In longitudinal sections of females at midbody, the ridges of *O. borneensis* n. sp. are half as high and half as wide at base as those of *O. d. dewittei* (Table 1; Fig. 2a, c). In addition, microfilariae of the new species are shorter than those of *O. d. dewittei* (Table 1). Furthermore, *O. borneensis* n. sp. can be differentiated from the other two remaining species of *Onchocerca* parasitizing suid hosts. These are *O. takaokai* Uni, Fukuda & Bain, 2015, described from *S. s. leucomystax* in Japan, whose females lack transverse cuticular ridges [18], and *O. ramachandrini* Bain, Wahl & Renz, 1993, described from *Phacochoerus africanus* (Gmelin) [as *P. aethiopicus* (Pallas)] in Cameroon, whose females lack transverse cuticular ridges but bear discontinuous longitudinal crests, and whose males possess inconspicuous transverse striations without short longitudinal crests at midbody and 11 pairs of caudal papillae [16].

**Prevalence, intensity and site in host**

*Onchocerca borneensis* n. sp. was found in two (ID nos. WB54 and WB68) of the three Bornean bearded pigs examined in the Long Banga area. These pigs were identified as *S. barbatus* by molecular methods (Additional file 2: Figure S1). The third pig (ID no. WB30) was small, probably one year-old. In the bearded pig WB54, one female worm was collected from the footpad of the forelimb, and six females and four males were obtained from nodular fibrous structures in the adipose tissue of two footpads of the hindlimbs. In the bearded pig WB68, one female and seven males were obtained from the footpad of the hindlimb. Many specimens were found in the hindlimbs. Several of the males collected were entangled with a female. We did not find microfilariae in the blood smears or skin snips of the limbs of either of the two infected bearded pigs.

***Onchocerca dewittei dewittei*** **from** ***Sus scrofa vittatus*** **in Peninsular Malaysia**

***Host***: *Sus scrofa vittatus* Boie (Cetartiodactyla: Suidae), banded pig.

***Locality***: Field Studies Center (3°19′29.0″N, 101°45′09.7″E) of the University of Malaya, situated in the primary forest of Ulu Gombak, Selangor, Peninsular Malaysia.

***Voucher material***: Specimens of *O. d. dewittei* were deposited in the Institute of Biological Sciences, University of Malaya, Malaysia (accession numbers: Od-F1–35 and Od-M1–2). Collection dates: 27.ii.2012, 14.xii.2012 and 20.v.2016.

***Site in host***: Adult worms were found in nodular fibrous structures in the adipose tissue of footpads of fore- and hindlimbs.

***Prevalence and intensity of infection***: Three of five banded pigs were infected with adult worms: 26 fragments of females and two entire males in WP1; one fragment of a female in WP4; and five fragments of females in WP5.

***Representative DNA sequences***: Sequence data were deposited in the GenBank database as follows: *cox*1 (MG209783, MG209785-MG209786); *12S* rRNA gene (MG209793-MG209798, MG973148); ITS (MG192128-MG192133, MK045758) for *O. d. dewittei*; *cyt*b (MG657266) for *S*. *s. vittatus*.

**Description**

***Female.*** [Based on 3 anterior parts, 9 midsections and 2 posterior parts; Table 1, Fig. 2c.] Transverse cuticular ridges spaced 40–93 apart at midbody. In longitudinal sections at midbody, ridges triangular, with pointed tip. Median layer of cuticle without internal striae (Fig. 2c). A single body swelling present at midbody.

***Male.*** [Based on 2 entire males; Table 1.] Apex of testis 2.8 mm from anterior extremity. Two body swellings at 5.3 mm and 14 mm from anterior extremity present in one male (45 mm long). Main part of body with small longitudinal cuticular crests.

***Microfilaria.*** [Based on 10 uterine microfilariae from fixed females and microfilariae from skin snips; Table 1.] Microfilariae from uteri unsheathed, not curled. Microfilariae from skin snips: body usually curled, 175–220 long, 5 wide, identified on the basis of morphological and molecular characteristics (data not shown).

**Prevalence, intensity and site in host**

Adult worms of *O. d. dewittei* were found in three (ID nos. WP1, WP4 and WP5) of five banded pigs (*S. s. vittatus*) in Peninsular Malaysia. The animals were captured in the primary forest of Ulu Gombak. The following entire worms and fragments were detected: three fragments of females in nodular fibrous structures in footpads of the forelimbs, 26 fragments of females and two entire males in footpads of the hindlimbs of WP1; one fragment of a female in the footpad of a forelimb of WP4; and five fragments of females in the footpads of the four limbs of WP5. As deduced from the number of teeth in wild boar, the animal (WP1) was 2.5 years-old. Four and five microfilariae of *O. d. dewittei* were found in skin snips taken from the limbs of WP1 and WP5, respectively. No microfilariae of *O. d. dewittei* were found in the skin snips of the remaining banded pigs. No microfilariae were found in the blood smears.

**Immunohistochemical staining of** ***Wolbachia*** **in the transverse sections**

*Wolbachia* symbionts were detected in the uteri and ovary but not in the lateral hypodermal chords or in the intestinal wall cells in a transverse section of a female *O. d. dewittei* (Fig. 2e).

***Onchocerca dewittei japonica*** **from** ***Sus scrofa leucomystax*** **in Japan**

***Host***: *Sus scrofa leucomystax* Temminck (Cetartiodactyla: Suidae), Japanese wild boar.

***Locality***: Bungoono (32°58′41.1″N, 131°35′06.2″E), Oita, Kyushu, Japan.

***Voucher material***: Specimens of *O. d. japonica* were deposited in the Institute for Research Promotion, Oita University, Japan (Accession numbers: Oj-F1–3 and Oj-M1–3). Collection date: 7.xi.2011.

***Site in host***: Adult worms were obtained from nodular fibrous structures in the footpads of fore- and hindlimbs.

***Prevalence and intensity of infection***: Both of two Japanese wild boars examined were infected with adult worms: two fragments of females and two fragments of males in OB4; two fragments of females and one fragment of a male in OB5.

***Representative DNA sequences***: Sequence data for *O. d. japonica* were deposited in the GenBank database as follows: *12S* rRNA gene (MG209799); ITS (MG192124).

***Female.*** [Based on 3 specimens; metrical data in Table 1.] Transverse cuticular ridges spaced 235–340 apart at midbody. In longitudinal sections at midbody, ridges triangular with wide base (Fig. 2d). Median layer of cuticle without internal striae.

***Male.*** [Based on 3 specimens; metrical data in Table 1.] Main part of body with small longitudinal cuticular crests.

***Microfilaria.*** [Based on 10 uterine microfilariae from fixed females.] Body 158–203 long, 5–6 wide.

### Morphological differentiation of *Onchocerca dewittei dewittei* and *O. d. japonica*

We confirm that the two subspecies differ in the distance between the transverse cuticular ridges as described previously [6]. Microfilariae of *O. d. dewittei* are slightly longer than those of *O. d. japonica* as reported by Uni et al. [6]. Moreover, we observed distinct differences in the shape of the ridges in longitudinal sections: the ridges of *O. d. dewittei* are one-third as high and one-fifth as wide at base as those of *O. d. japonica* (Table 1; Fig. 2c, d). Furthermore, females of *O. d. dewittei* are narrower at midbody than those of *O. d. japonica* (Table 1). The above characteristics can be used to morphologically distinguish *O. d. japonica* from *O. d. dewittei*.

### Molecular analyses

**Fig. 3:**
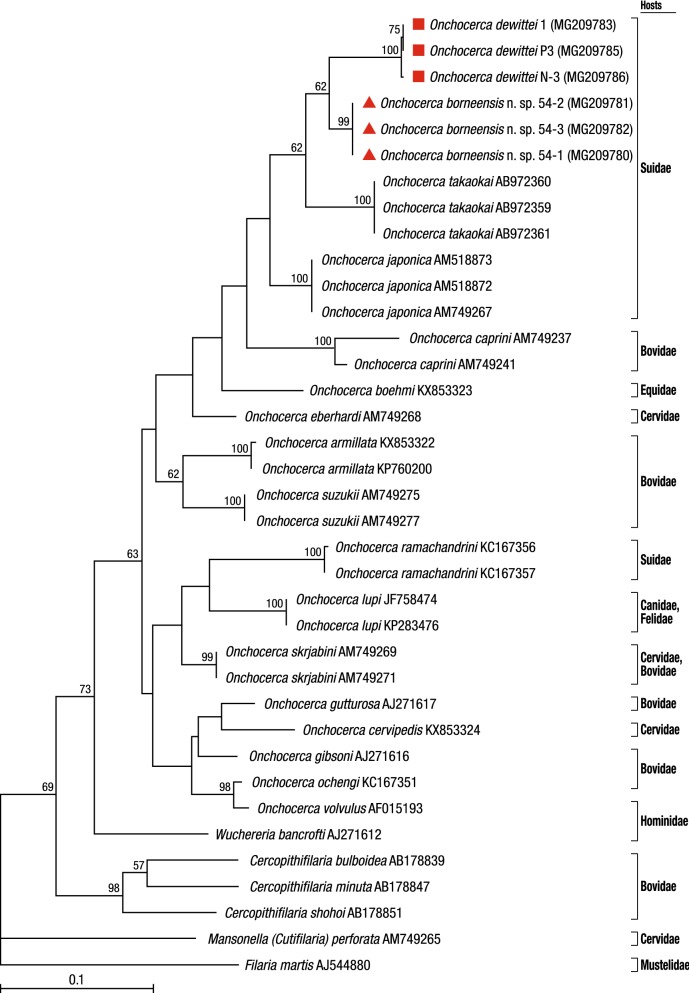
Taxonomic position of *Onchocerca borneensis* n. sp. and *O. dewittei* based on *cox*1 nucleotide sequences. The maximum-likelihood phylogenetic tree was generated under the Tamura-Nei model in MEGA7 with 500 bootstrap replicates. The scale-bar below the diagram indicates the number of inferred changes along each branch. Red triangles and squares indicate sequences generated in this study

**Fig. 4:**
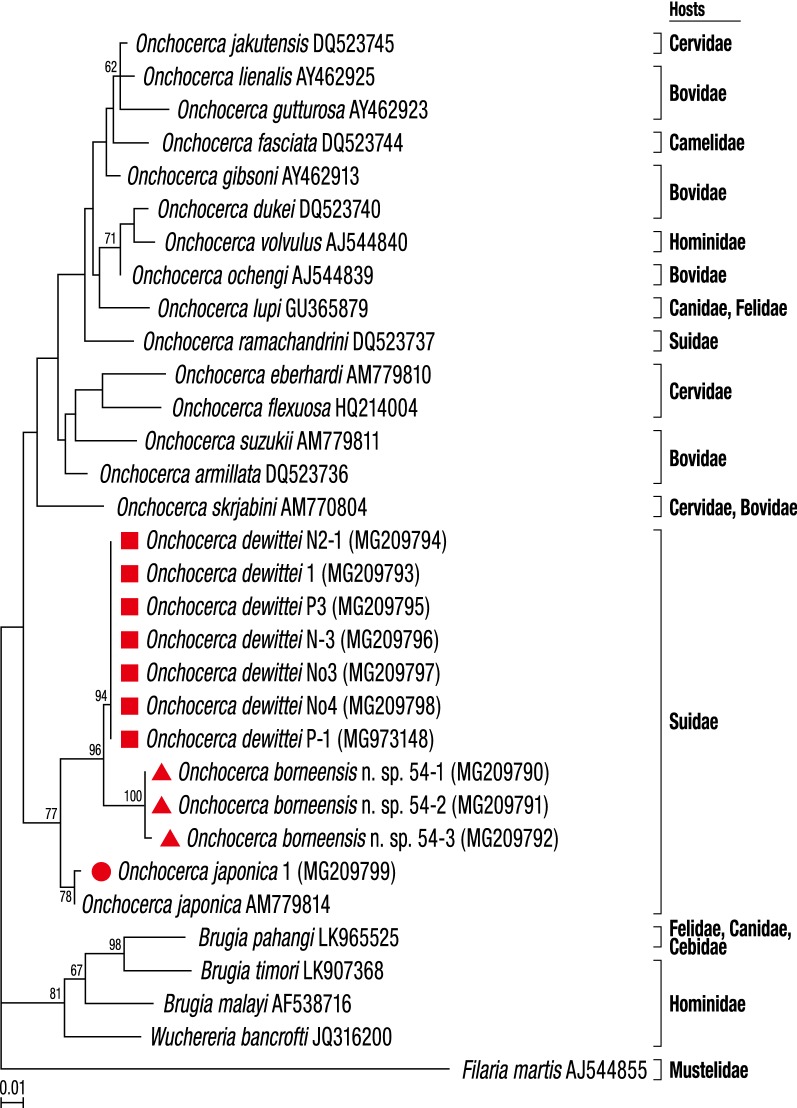
Taxonomic position of *Onchocerca borneensis* n. sp., *O. dewittei* and *O. japonica* based on *12S* rDNA nucleotide sequences. The maximum-likelihood phylogenetic tree was generated under the Tamura-Nei model in MEGA7 with 500 bootstrap replicates. The scale-bar below the diagram indicates the number of inferred changes along each branch. Red triangles, squares and circle indicate sequences generated in this study

**Fig. 5:**
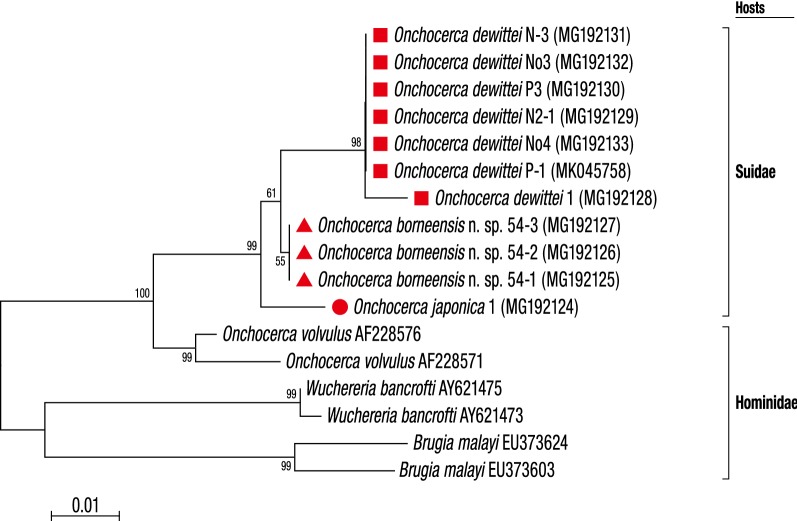
Taxonomic position of *Onchocerca borneensis* n. sp., *O. dewittei* and *O. japonica* based on ITS nucleotide sequences. The maximum-likelihood phylogenetic tree was generated under the General Time Reversible model in MEGA7 with 500 bootstrap replicates. The scale-bar below the diagram indicates the number of inferred changes along each branch. Red triangles, squares and circle indicate sequences generated in this study

To elucidate the evolutionary relationships between *O. borneensis* n. sp., *O. d. dewittei*, *O. d. japonica*, *O. takaokai* and *O. ramachandrini* from suid hosts, we compared their *cox*1, *12S* rDNA and ITS sequences with those of other filarial parasites available on GenBank. Calculated p-distances for the *cox*1 sequences between *O. borneensis* n. sp. and its congeners were 5.9% for *O. d. dewittei*, 6.4% for *O. takaokai*, 6.9% for *O. d. japonica* and 11.5–11.7% for *O. ramachandrini*. The p-distance for the *cox*1 sequences between *O. d. dewittei* and *O. d. japonica* was 7.6% (Additional file 1: Table S1). According to Ferri et al. [50], filarioid nematodes can be considered different species if the genetic distance based on the *cox*1 sequences is greater than 4.8%. In *Onchocerca* spp., *cox*1 interspecific distances are higher than 4.5% and intraspecific distances are lower than 2% [20]. Therefore, the molecular findings corroborate the morphological data, further supporting *O. borneensis* n. sp. as a species distinct from *O. d. dewittei* described in Peninsular Malaysia and other congeners. Moreover, on the basis of the morphological differences between *O. d. dewittei* and *O. d. japonica* set out above and the high molecular divergence between these two taxa, we propose to elevate *O. d. japonica* to species level as *O. japonica* Uni, Bain & Takaoka, 2001. In the phylogenetic trees based on ML inference using sequence data from the two mitochondrial genes, *cox*1 and *12S* rRNA, as well as the nuclear ITS region, *O. borneensis* n. sp. was placed as a sister species to *O. dewittei* from Suidae (Figs. 3, 4 and 5).

The molecular characteristics of two bearded pigs (WB54 and WB68) in Borneo and a banded pig (WP5) in Peninsular Malaysia are presented in Additional file 2: Figure S1. The two host animals in Borneo were identified as *S. barbatus* by the present molecular analysis, whereas the host animal at Ulu Gombak in Selangor, Peninsular Malaysia, was morphologically identified as *S. s. vittatus.* Sequence data for comparison with *S. s. vittatus* from other studies were not available. The host animal from Peninsular Malaysia and *S. barbatus* from Borneo occupy vastly different positions in the present phylogenetic tree (Additional file 2: Figure S1).

### *Wolbachia* infection

**Fig. 6:**
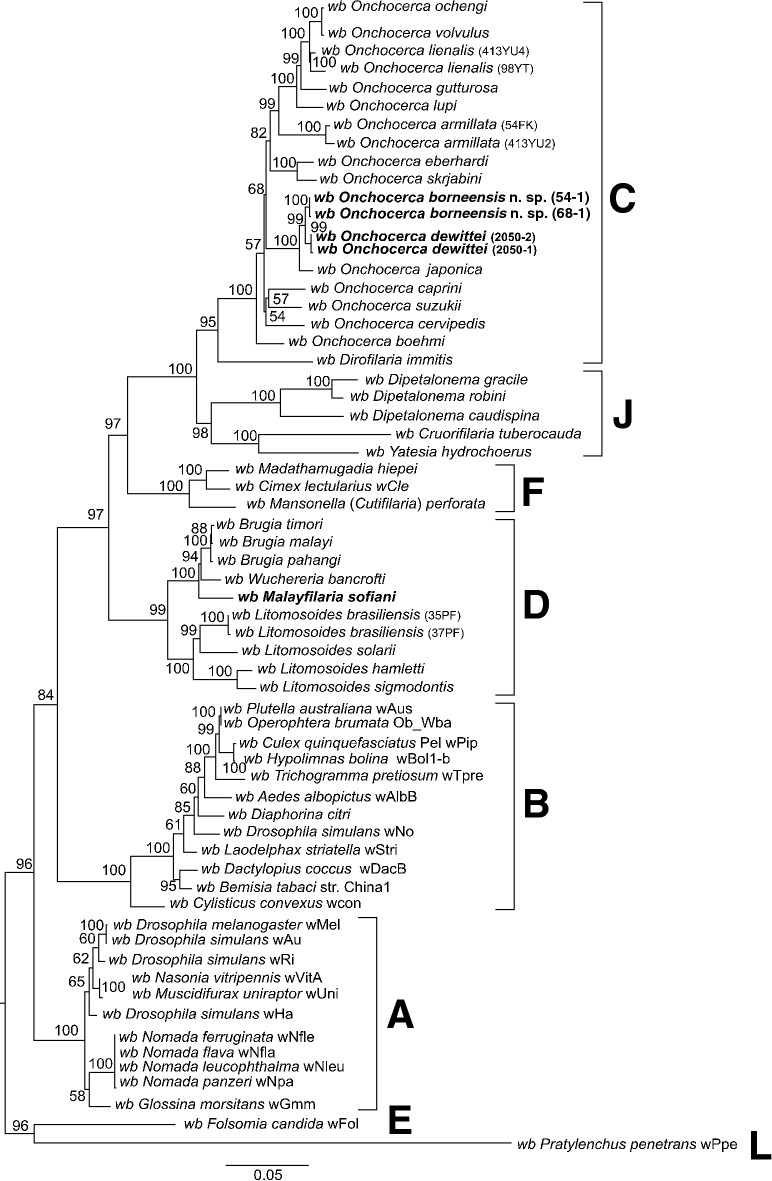
Phylogenetic tree of *Wolbachia* based on six markers using maximum-likelihood inference. Analysis based on concatenation of *16S* rDNA, *ftsZ*, *dnaA*, *coxA*, *fbpA* and *gatB* (3086 bp in total length). The topology was generated using model TIM + F + I + G4 with IQ-TREE. The robustness of nodes was assessed with 1000 bootstrap replicates. The *Wolbachia* supergroups (A–F, J and L) were identified according to Lefoulon et al. [28] and Lo et al. [43]. The scale-bar indicates the distance in substitutions per nucleotide. *Abbreviation*: *wb, Wolbachia*

*Onchocerca borneensis* n. sp. and *O. dewittei* both harboured *Wolbachia* strains belonging to supergroup C (Fig. 6). The *Wolbachia* strains in *O. borneensis* n. sp. were closely related to the strains in *O. dewittei*. *Malayfilaria sofiani* harboured a strain of *Wolbachia* supergroup D; this strain was placed in a sister position to the clade formed by strains of *W. bancrofti* and species of *Brugia*.

## Discussion

Based on their morphological characteristics, *O. borneensis* n. sp., *O. dewittei* and *O. japonica* constitute a sub-group within the five species of *Onchocerca* parasitizing hosts of the Suidae. Their predilection sites in the host as well as the hosts themselves (*Sus* Linnaeus) are equally similar. In contrast, *O. ramachandrini*, whose females possess longitudinal, not transverse, cuticular crests, is not only morphologically distinct from the above sub-group (Table 1), but its host, *P. africanus* in the Afrotropics [16], is markedly different from the hosts (*Sus*) of the four *Onchocerca* species in Asia. The host genus *Phacochoerus* F. Cuvier belongs to Phacochoerini, a distinct tribe within the Suinae [51].

Molecular analyses based on *cox*1, *12S* rDNA and ITS sequences indicated that *O. borneensis* n. sp. and *O. dewittei* form a monophyletic clade (Figs. 3, 4 and 5). *Onchocerca takaokai* from Japan was close to the clade in the *cox*1 tree (Fig. 3) and also *O. japonica* from Japan was close to the clade in other trees (Figs. 5, 6). Furthermore, the four species (*O. dewittei*, *O. borneensis* n. sp., *O. takaokai* and *O. japonica*) parasitic in modern pigs (*Sus*) were closely related in the *cox*1 tree (Fig. 3) and based on the genetic distances (Additional file 1: Table S1). It is interesting to note that *O. borneensis* n. sp. and *O. dewittei* infect *S. barbatus* and *S. s. vittatus*, respectively, in the Indomalayan realm, but *O. japonica* and *O. dewittei* infect different subspecies of the same host species (*S. scrofa* Linnaeus) in the Palaearctic realm and the Indomalayan realm, respectively. This fact suggests that the host biogeographical area is a crucial factor for *Onchocerca* diversification. Conversely, the genetic distance between *O. borneensis* n. sp. and *O. ramachandrini* was pronounced and ranged from 11.5–11.7% (Additional file 1: Table S1). Krueger et al. [52] suggested that *O. ramachandrini* is one of the ancestral lineages in the genus *Onchocerca*.

Considering the evolutionary history of the Suidae, the ancestor of modern pigs (*Sus*) has been separated from the ancestor of sub-Saharan suids, including warthogs (*Phacochoerus*), since the late Miocene (> 10 Ma) [53–55]. During the late Pliocene/early Pleistocene (2.5 Ma), the genus *Sus* arose in the tropical inland Southeast Asia (ISEA) and subsequently diversified into multiple species. While *S. barbatus* and several other species are restricted to the ISEA, *S. scrofa* expanded from Southeast Asia to the whole of Eurasia and North Africa [54–57]. Our molecular analysis identified *S. barbatus* as host of *O. borneensis* n. sp. in Borneo (Additional file 2: Figure S1). In Peninsular Malaysia, we morphologically identified *S. s. vittatus* as host of *O. dewittei*, on the basis of the absence of a beard. Except for the data generated in the present study, no sequence data for *S. s. vittatus* are available. Our molecular analysis indicates that the banded pigs from Peninsular Malaysia differ from *S. barbatus* and *S. verrucosus* Boie. The results obtained for *Sus* species in the present study generally resemble the phylogeny of the Suinae proposed by Frantz et al. [54, 55]. We thus speculate that *Onchocerca* species in the Suidae, in conjunction with the diversification of their hosts, diversified into two lineages: one ancestral lineage leading to *O. ramachandrini* in the African Suinae, and another ancestral lineage leading to *O. borneensis* n. sp. and *O. dewittei* when *Sus* diversified in the ISEA. With regard to the origin of *O. japonica*, Lefoulon et al. [20] proposed the transfer of *L. caprini* Uni & Bain, 2006 to the genus *Onchocerca* and suggested that *O. japonica* arose from the ancestral lineage of *O. caprini* following a host switch from Caprinae to Suidae.

In sections of *O. dewittei* examined by immunostaining in the present study (Fig. 2e), *Wolbachia* symbionts were detected in the female germ line, but not in the lateral chords or intestinal wall. In *O. japonica*, *Wolbachia* was found in the intra-uterine ova and ovaries in transverse sections [34]. Although not detected in the lateral chords in these sections, a few *Wolbachia* were observed in the lateral chords when using whole mount fluorescent analysis [34, 58]. It is thus difficult to decide if the absence of *Wolbachia* in the lateral chords of *O. dewittei* in the present study reflects a true absence or the limitations of our methodology. Among several bacterial strains identified as *Wolbachia* supergroups, supergroups C, D, F and J have been detected in the Onchocercidae [28, 31, 34]. These supergroups have been detected in the female germline and the lateral hypodermal chords in many filarioid nematodes by immunostaining and/or whole-mount fluorescent analysis [28, 34, 35].

With regard to the phylogenetic relationships between *Wolbachia* supergroups and clades (ONC1–ONC5) of Onchocercidae, horizontal transfer events of *Wolbachia*, secondary losses and local coevolution with host filarioid nematodes are discussed [28, 34, 35]. Our molecular analyses indicate that the *Wolbachia* strains in *O. borneensis* n. sp. and *O. dewittei* form the sister group and are closely related to the strain of *O. japonica* (Fig. 6). In other words, the molecular relationships of these *Wolbachia* strains are congruent with the local molecular pattern of their host filarioid nematodes (*O. borneensis* n. sp. and *O. dewittei*) from Asian suids (Figs. 3, 4 and 5). Our results support the notion of a coevolutionary pattern between supergroup C strains and species of ONC3, as proposed by Lefoulon et al. [20, 28]. In addition, the *Wolbachia* strain infecting *M. sofiani* belongs to supergroup D and is closely related to *Wolbachia* strains in *W. bancrofti* and *Brugia* spp. of ONC5 (Fig. 6). Thus, we also found a coevolutionary pattern between these *Wolbachia* strains and their respective host filarioid nematodes. Close molecular relationships between *M. sofiani*, *W. bancrofti* and *Brugia* spp. have previously been proposed by Uni et al. [24].

Human infections caused by *O. japonica* have recently been reported in Japan [7, 8], and the prevalence of *O. japonica* in *S. s. leucomystax* was found to be 89% in Oita, Japan [59]. In this study, we found *O. borneensis* n. sp. from *S. barbatus* in Borneo. Consequently, there is a potential for zoonotic infection by *O. borneensis* n. sp. and *O. dewittei* to occur, given that various species within the genus are known to cause zoonotic infection.

## Conclusions

We described *O. borneensis* n. sp. found in the footpads of bearded pigs (*S. barbatus*) in Borneo. The new species differs from *O. dewittei* in banded pigs of Peninsular Malaysia in several morphological characteristics. According to molecular analyses, the *cox*1 gene sequence of *O. borneensis* n. sp. differs from that of *O. dewittei* by 5.9%. Taking into consideration both morphological characteristics and genetic divergence, we conclude that *O. borneensis* n. sp. is most closely related to *O. dewittei* from Peninsular Malaysia. Furthermore, because *cox*1 sequences of *O. japonica* and *O. dewittei* differ by 7.6%, we consider that *O. japonica* is a distinct species, separate from *O. dewittei*. We detected *Wolbachia* supergroup C strains in *O. borneensis* n. sp. and *O. dewittei*, confirming a coevolutionary pattern between *Wolbachia* strains and their host *Onchocerca* spp. from Asian suids.

## Supplementary information

**Additional file 1: Table S1.** Uncorrected p-distances for the *cox*1 gene sequences between *Onchocerca borneensis* n. sp., *O. dewittei* and other known filarial species.

**Additional file 2: Figure S1.** Taxonomic position of *Sus barbatus* and *S. scrofa vittatus* based on *cyt*b nucleotide sequences. The maximum-likelihood phylogenetic tree was generated under the General Time Reversible model in MEGA7 with 500 bootstrap replicates. The scale-bar below the diagram indicates the number of inferred changes along each branch. Red triangles and square indicate sequences generated in this study.

**Additional file 3: Table S2.** GenBank accession numbers for endosymbiont *Wolbachia*. Accession numbers in bold (*Malayfilaria sofiani*, *Onchocerca borneensis* n. sp. and *O. dewittei*) represent sequences produced for the present study. *Abbreviations*: ext., samples from other studies; Ø, no sequences.

## Data Availability

The data supporting the conclusions of this article are included within the article and its additional files. The holotype and allotype of *O. borneensis* n. sp. were deposited in the MNHN, Paris, France, under accession numbers MNHN 103YT and 104YT, and the paratypes were deposited in the Institute of Biological Sciences, University of Malaya, Malaysia, under accession numbers Ob-B54-1–2, Ob-B54-4, Ob-B54-6–7, Ob-54-9, Ob-B68-2, Ob-B54-2M1–2, Ob-B54-3M1 and Ob-B68-2-M3–9. Specimens of *O. dewittei* were deposited in the Institute of Biological Sciences, University of Malaya, Malaysia, under accession numbers Od-F1–﻿35 and Od-M1–2. Specimens of *O. japonica* were deposited in the Institute for Research Promotion, Oita University, Japan, under accession numbers Oj-F1-3 and Oj-M1-﻿3. Sequences were deposited in the GenBank database under the accession numbers: *cox*1 (MG209780-MG209782), *12S* rRNA gene (MG209790-MG209792), ITS (MG192125-MG192127) for *O. borneensis* n. sp.; *cyt*b (MG657264-MG657265) for *S. barbatus*; c*ox*1 (MG209783, MG209785-MG209786), *12S* rRNA gene (MG209793-MG209798, MG973148), ITS (MG192128-MG192133, MK045758) for *O. dewittei*; *cyt*b (MG657266) for *S. s. vittatus*; *12S* rRNA gene (MG209799), ITS (MG192124) for *O. japonica*. Data for *Wolbachia* are provided in Additional file 3: Table S2.

## Abbreviations

*cox*1: cytochrome *c* oxidase subunit 1 gene
*cyt*b: cytochrome *b* gene
HE: haematoxylin and eosin
ISEA: inland Southeast Asia
ITS: internal transcribed spacer
Ma: mega annum
ML: maximum-likelihood
MNHN: Muséum National d’Histoire Naturelle
CIH: Commonwealth Institute of Helminthology
PCR: polymerase chain reaction
*wb*: *Wolbachia*
