# Supplementary material for: Description, molecular characteristics and Wolbachia endosymbionts of Onchocerca borneensis Uni, Mat Udin & Takaoka n. sp. (Nematoda: Filarioidea) from the Bornean bearded pig Sus barbatus Müller (Cetartiodactyla: Suidae) of Sarawak, Malaysia
Source: Parasit Vectors. 2020 Feb 6;13:50. doi: 10.1186/s13071-020-3907-8 (PMC7006428; doi:10.1186/s13071-020-3907-8)
Supplement: Supplementary file 1 — Additional file 1: Table S1. Uncorrected p-distances for the cox1 gene sequences between Onchocerca borneensis n. sp., O. dewittei and other known filarial species. [file 13071_2020_3907_MOESM1_ESM.pdf]

Additional file 1. Table S1 Uncorrected p-distances for the *cox*1 gene sequences between *Onchocerca borneensis* n. sp., *O. dewittei* and other known filarial species

		1	2	3	4	5	6	7	8	9	10	11	12	13	14	15	16	17	18	19	20	21	22	23
1	*Onchocerca borneensis* n. sp. 54-1 (MG209780)																							
2	*Onchocerca borneensis* n. sp. 54-2 (MG209781)	0.000																						
3	*Onchocerca borneensis* n. sp. 54-3 (MG209782)	0.000	0.000																					
4	*Onchocerca dewittei* P3 (MG209785)	0.059	0.059	0.059																				
5	*Onchocerca dewittei* 1 (MG209783)	0.059	0.059	0.059	0.000																			
6	*Onchocerca dewittei* N-3 (MG209786)	0.059	0.059	0.059	0.003	0.003																		
7	*Onchocerca takaokai* AB972359	0.064	0.064	0.064	0.089	0.089	0.089																	
8	*Onchocerca takaokai* AB972360	0.064	0.064	0.064	0.089	0.089	0.089	0.000																
9	*Onchocerca takaokai* AB972361	0.064	0.064	0.064	0.089	0.089	0.089	0.000	0.000															
10	*Onchocerca japonica* AB518872	0.069	0.069	0.069	0.076	0.076	0.076	0.084	0.084	0.084														
11	*Onchocerca japonica* AB518873	0.069	0.069	0.069	0.076	0.076	0.076	0.084	0.084	0.084	0.000													
12	*Onchocerca japonica* AM749267	0.069	0.069	0.069	0.076	0.076	0.076	0.084	0.084	0.084	0.000	0.000												
13	*Onchocerca ochengi* KC167351	0.081	0.081	0.081	0.094	0.094	0.094	0.097	0.097	0.097	0.104	0.104	0.104											
14	*Onchocerca volvulus* AF015193	0.084	0.084	0.084	0.097	0.097	0.097	0.099	0.099	0.099	0.102	0.102	0.102	0.015										
15	*Onchocerca gibsoni* AJ271616	0.092	0.092	0.092	0.109	0.109	0.109	0.087	0.087	0.087	0.109	0.109	0.109	0.056	0.064									
16	*Onchocerca gutturosa* AJ271617	0.097	0.097	0.097	0.102	0.102	0.102	0.112	0.112	0.112	0.099	0.099	0.099	0.064	0.064	0.059								
17	*Onchocerca ramachandrini* KC167356	0.115	0.115	0.115	0.137	0.137	0.137	0.127	0.127	0.127	0.109	0.109	0.109	0.112	0.115	0.104	0.112							
18	*Onchocerca ramachandrini* KC167357	0.117	0.117	0.117	0.140	0.140	0.140	0.125	0.125	0.125	0.107	0.107	0.107	0.115	0.117	0.102	0.109	0.003						
19	*Wuchereria bancrofti* AJ271612	0.125	0.125	0.125	0.137	0.137	0.137	0.117	0.117	0.117	0.127	0.127	0.127	0.120	0.122	0.099	0.115	0.130	0.127					
20	*Cercopithifilaria minuta* AB178847	0.150	0.150	0.150	0.165	0.165	0.165	0.153	0.153	0.153	0.140	0.140	0.140	0.132	0.135	0.140	0.132	0.176	0.173	0.132				
21	*Cercopithifilaria bulboidea* AB178839	0.163	0.163	0.163	0.165	0.165	0.165	0.155	0.155	0.155	0.158	0.158	0.158	0.148	0.148	0.158	0.168	0.173	0.170	0.145	0.104			
22	*Cercopithifilaria shohoi* AB178851	0.168	0.168	0.168	0.170	0.170	0.170	0.170	0.170	0.170	0.165	0.165	0.165	0.165	0.165	0.168	0.158	0.181	0.178	0.150	0.120	0.112		
23	*Mansonella perforata* AM749265	0.178	0.178	0.178	0.183	0.183	0.183	0.170	0.170	0.170	0.188	0.188	0.188	0.165	0.163	0.170	0.165	0.198	0.196	0.178	0.183	0.191	0.181	
24	*Filaria martis* AJ544880	0.186	0.186	0.186	0.201	0.201	0.201	0.193	0.193	0.193	0.188	0.188	0.188	0.206	0.201	0.204	0.191	0.211	0.209	0.206	0.209	0.201	0.193	0.211
