# Supplementary material for: Description, molecular characteristics and Wolbachia endosymbionts of Onchocerca borneensis Uni, Mat Udin & Takaoka n. sp. (Nematoda: Filarioidea) from the Bornean bearded pig Sus barbatus Müller (Cetartiodactyla: Suidae) of Sarawak, Malaysia
Source: Parasit Vectors. 2020 Feb 6;13:50. doi: 10.1186/s13071-020-3907-8 (PMC7006428; doi:10.1186/s13071-020-3907-8)
Supplement: Supplementary file 2 — Additional file 2: Figure S1. Taxonomic position of Sus barbatus and S. scrofa vittatus based on cytb nucleotide sequences. The maximum-likelihood phylogenetic tree was generated under the General Time Reversible model in MEGA7 with 500 bootstrap replicates. The scale-bar below the diagram indicates the number of inferred changes along each branch. Red triangles and square indicate sequences generated in this study. [file 13071_2020_3907_MOESM2_ESM.pdf]

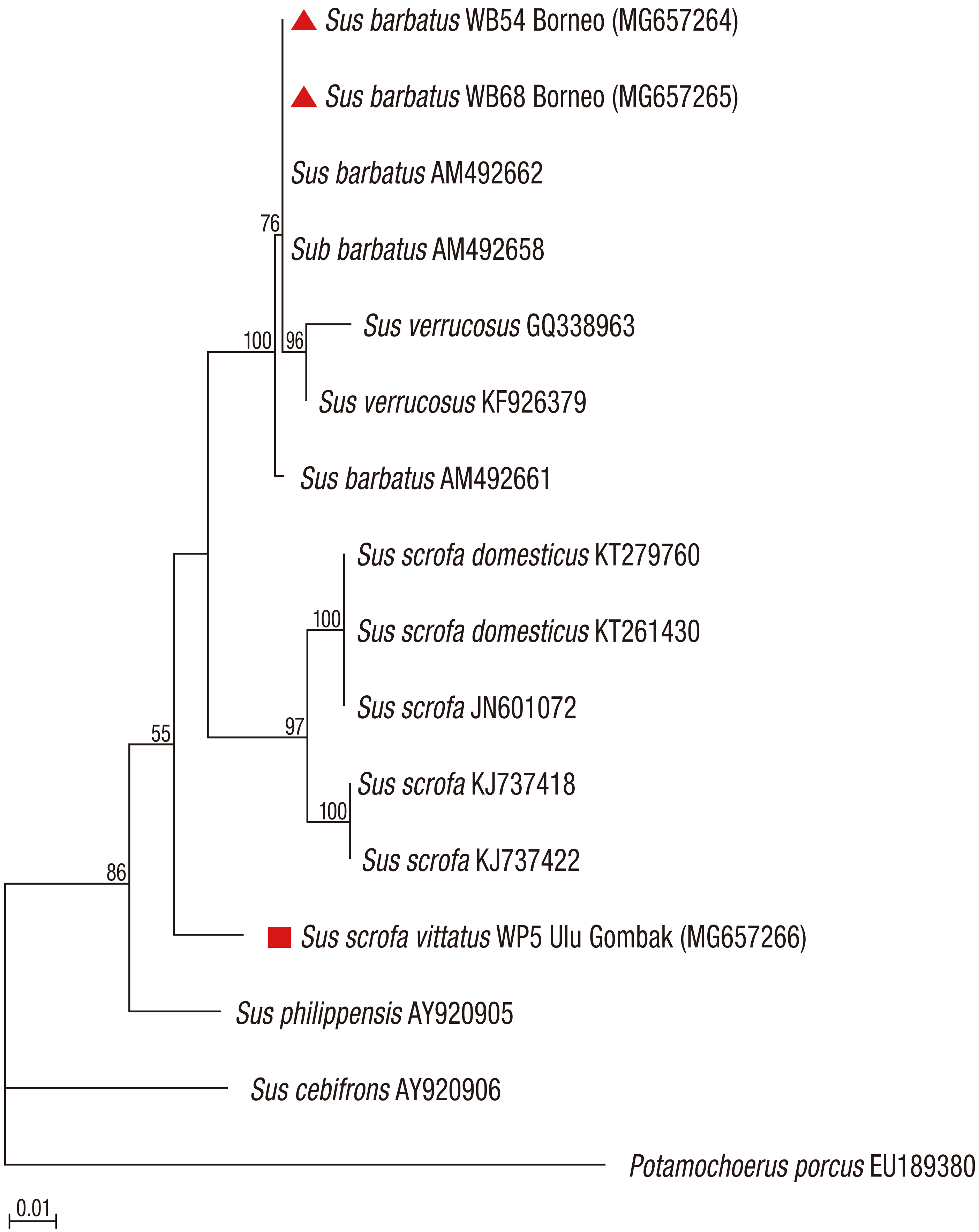

Additional file 2: Figure S1. Taxonomic position of *Sus barbatus and S. scrofa vittatus* based on *cytb* nucleotide sequences. The maximum-likelihood phylogenetic tree was generated under the General Time Reversible model in MEGA7 with 500 bootstrap replicates. The scale-bar below the diagram indicates the number of inferred changes along each branch. Red triangles and square indicate sequences generated in this study.
