# Supplementary material for: Description, molecular characteristics and Wolbachia endosymbionts of Onchocerca borneensis Uni, Mat Udin & Takaoka n. sp. (Nematoda: Filarioidea) from the Bornean bearded pig Sus barbatus Müller (Cetartiodactyla: Suidae) of Sarawak, Malaysia
Source: Parasit Vectors. 2020 Feb 6;13:50. doi: 10.1186/s13071-020-3907-8 (PMC7006428; doi:10.1186/s13071-020-3907-8)
Supplement: Supplementary file 3 — Additional file 3: Table S2. GenBank accession numbers for endosymbiont Wolbachia. Accession numbers in bold (Malayfilaria sofiani, Onchocerca borneensis n. sp. and O. dewittei) represent sequences produced for the present study. Abbreviations: ext., samples from other studies; Ø, no sequences. [file 13071_2020_3907_MOESM3_ESM.pdf]

**Additional file 3. Table S2** GenBank accession numbers for endosymbiont *Wolbachia*

Accession numbers in bold (*Malayfilaria sofiani*, *Onchocerca borneensis* n. sp. and *O. dewittei*) represent sequences produced for the present study. *Abbreviations*: ext., samples from other studies; Ø, no sequences

| | | | | ***Wolbachia*** | | | | | |
|---|---|---|---|---|---|---|---|---|---|
| | **Host** | **Strain** | **Sample ID** | **16S rDNA** | ***ftsZ*** | ***dnaA*** | ***coxA*** | ***fbpA*** | ***gatB*** |
| NEMATODES | *Brugia malayi* | | ext. | NC_006833 | | | | | |
| | *Brugia pahangi* | | 46YT | KU255226 | KU255348 | KU255289 | KU255257 | KU255319 | KU255369 |
| | *Brugia timori* | | 6YT | KU255227 | KU255349 | KU255290 | KU255258 | KU255320 | KU255370 |
| | *Cruorifilaria tuberocauda* | | 55YT | KU255229 | Ø | KU255292 | KU255260 | KU255322 | KU255372 |
| | *Dipetalonema caudispina* | | 362YU | KU255230 | Ø | KU255293 | KU255261 | KU255323 | KU255373 |
| | *Dipetalonema gracile* | | 124CV | KU255233 | Ø | KU255296 | KU255264 | KU255325 | KU255375 |
| | *Dipetalonema robini* | | 217YU | KU255237 | Ø | KU255301 | KU255269 | KU255329 | KU255377 |
| | *Dirofilaria immitis* | | ext. | wDi.2.2 | | | | | |
| | *Litomosoides brasiliensis* | | 35PF | KU255241 | KU255354 | KU255305 | KU255273 | KU255333 | KU255381 |
| | *Litomosoides brasiliensis* | | 37PF | KU255242 | KU255355 | KU255306 | KU255274 | KU255334 | KU255382 |
| | *Litomosoides hamletti* | | PF36 | KU255244 | KU255356 | KU255308 | KU255276 | KU255335 | KU255384 |
| | *Litomosoides sigmodontis* | | ext. | wLs.2.2 | | | | | |
| | *Litomosoides solarii* | | 213YU | KU255245 | KU255358 | KU255309 | KU255277 | KU255336 | KU255385 |
| | *Madathamugadia hiepei* | | 81YU | JQ888303 | JQ888330 | JQ888321 | JQ888307 | JQ888342 | KU255386 |
| | ***Malayfilaria sofiani*** | | **KE-2** | **MN319534** | **MN313598** | **MN313602** | Ø | **MN313594** | **MN313607** |
| | *Mansonella (Cutifilaria) perforata* | | 216JW | KU255246 | KU255360 | FR827919 | KU255278 | KU255337 | KU255387 |
| | *Onchocerca armillata* | | 54FKa1 | KU255247 | KU255361 | KU255310 | KU255280 | KU255338 | KU255388 |
| | *Onchocerca armillata* | | 413YU2 | KX853427 | KX853410 | KX853401 | KX853393 | KX853384 | KX853376 |
| | *Onchocerca boehmi* | | 88YT | KX853428 | KX853411 | KX853402 | KX853394 | KX853385 | KX853377 |
| | ***Onchocerca borneensis* n. sp.** | | **54-1** | **MN319533** | **MN313597** | Ø | **MN313589** | **MN313593** | **MN313606** |
| | ***Onchocerca borneensis* n. sp.** | | **68-1** | **MN319532** | **MN313596** | **MN313601** | **MN313588** | **MN313592** | **MN313605** |
| | *Onchocerca caprini* | | YG2-58 | KU255243 | FR827922 | KU255307 | KU255275 | KX853391 | KU255383 |
| | *Onchocerca cervipedis* | | 97YT | KX853429 | KX853419 | KX853409 | KX853400 | KX853392 | KX853383 |
| | ***Onchocerca dewittei*** | | **2050-1** | **MN319531** | **MN313595** | **MN313600** | **MN313587** | **MN313591** | **MN313604** |
| | ***Onchocerca dewittei*** | | **2050-2** | **MN319530** | Ø | **MN313599** | **MN313586** | **MN313590** | **MN313603** |
| | *Onchocerca japonica* | | OB9 | KU255250 | KU255363 | KU255313 | KU255283 | KU255341 | KU255391 |
| | *Onchocerca eberhardi* | | S63-5 | KU255248 | KX853418 | KU255311 | KU255281 | KU255339 | KU255389 |
| | *Onchocerca lienalis* | | 413YU4 | KX853430 | KX853416 | KX853407 | KX853398 | KX853388 | KX853381 |
| | *Onchocerca lienalis* | | 98YT | KX853431 | KX853417 | KX853408 | KX853399 | KX853389 | KX853382 |
| | *Onchocerca lupi* | | 88YTA | KX853434 | KX853414 | KX853405 | KX853396 | KX853386 | KX853379 |
| | *Onchocerca gutturosa* | | 54FKg1 | KU255249 | KU255362 | KU255312 | KU255282 | KU255340 | KU255390 |
| | *Onchocerca ochengi* | | ext. | NC_018267 | | | | | |
| | *Onchocerca skrjabini* | | S63-6 | KU255252 | KU255365 | KU255315 | KU255285 | KU255343 | KU255393 |
| | *Onchocerca suzukii* | | S63-8 | KX853435 | KX853415 | KX853406 | KX853397 | KX853390 | KX853380 |
| | *Onchocerca volvulus* | | ext. | NZ_HG810405 | | | | | |
| | *Pratylenchus penetrans* | *wPpe* | ext. | NZ_MJMG01000000 | | | | | |
| | *Wuchereria bancrofti* | | ext. | NZ_NJBR02000000 | | | | | |
| | *Yatesia hydrochoerus* | | 52YT | KU255254 KU255255 | Ø | KU255317 | KU255286 KU255287 | KU255345 KU255346 | KU255395 |
| ARTHROPODS | *Aedes albopictus* | *wAlbB* | ext. | NZ_CP031221 | | | | | |
| | *Bemisia tabaci* | *China 1* | ext. | NZ_CP016430 | | | | | |
| | *Cimex lectularius* | *wCle* | ext. | NZ_AP013028 | | | | | |
| | *Culex quinquefasciatus Pel* | *wPip* | ext. | NC_010981 | | | | | |
| | *Cylisticus convexus* | *Wcon* | ext. | NZ_QPIP01000000 | | | | | |
| | *Dactylopius coccus* | *wDacB* | ext. | NZ_LSYY01000000 | | | | | |
| | *Diaphorina citri* | *wACP3* | ext. | NZ_KB223536 | | | | | |
| | *Drosophila melanogaster* | *wMel* | ext. | NC_002978 | | | | | |
| | *Drosophila simulans* | *wAu* | ext. | NZ_LK055284 | | | | | |
| | *Drosophila simulans* | *wHa* | ext. | NC_021089 | | | | | |
| | *Drosophila simulans* | *wNo* | ext. | NC_021084 | | | | | |
| | *Drosophila simulans* | *wRi* | ext. | NC_012416 | | | | | |
| | *Folsomia candida* | *wFol* | ext. | NZ_CP015510 | | | | | |
| | *Glossina morsitans* | *wGmm* | ext. | NZ_AWUH01000000 | | | | | |
| | *Hypolimnas bolina* | *wBol1-b* | ext. | NZ_CAOH01000000 | | | | | |
| | *Muscidifurax uniraptor* | *wUni* | ext. | NZ_MUJL01000000 | | | | | |
| | *Nasonia vitripennis* | *wVitA* | ext. | NZ_MUJM01000000 | | | | | |
| | *Nomada ferruginata* | *wNfe* | ext. | NZ_LYUY01000000 | | | | | |
| | *Nomada flava* | *wNfla* | ext. | NZ_LYUW01000000 | | | | | |
| | *Nomada leucophthalma* | *wNleu* | ext. | NZ_LYUV01000000 | | | | | |
| | *Nomada panzeri* | *wNpa* | ext. | NZ_LYUX01000000 | | | | | |
| | *Laodelphax striatella* | *wStri* | ext. | NZ_LRUH01000000 | | | | | |
| | *Operophtera brumata* | *Ob_Wba* | ext. | NZ_JYPC01000000 | | | | | |
| | *Plutella australiana* | *wAus* | ext. | NZ_MRWX01000000 | | | | | |
| | *Trichogramma pretiosum* | *wTpre* | ext. | NZ_CM003641 | | | | | |
